# Dignity as Sovereignty

**DOI:** 10.1017/jme.2025.10212

**Published:** 2026

**Authors:** Jennifer Hardes Dvorak

**Affiliations:** Law, Canterbury Christ Church University, United Kingdom

**Keywords:** dignity, sovereignty, personhood

## Abstract

This commentary suggests that the meaning and content of dignity is bound to the broader question of who is said to have personhood and sovereignty, and thus protection and rights under the law, and who is excluded from our legal community.

Scarffe’s article^1^ raises an interesting question about how the concept of dignity is invoked in legal cases concerning the beginning of life. In particular, Scarffe questions the notion that dignity is a settled moral concept and instead conceives of dignity as a legal status that is conferred in context. As he notes, cases do not always replicate the same dominant meaning of dignity, which is rehearsed in the form of a Kantian or even capabilities approach to dignity, both of which locate dignity in the autonomy of the person. Instead, Scarffe sees the legally generated meaning of dignity as sometimes leaning on this dominant idea and sometimes departing from it in favor of other conceptions of dignity.

Drawing on the judgment of Justice Kennedy in *Ohio v. Akron Center*, 497 U.S. 502 (1990), Scarffe argues that Kennedy pulled dignity “in the other direction” from earlier articulations of women’s dignity that had been understood as the right to make choices regarding abortion free from government interference, seen in *Thornburgh v. American College of Obstetricians*, 476 U.S. 747 (1986). Scarffe also argues that in *Akron*, laws in Ohio requiring physicians to notify parents in cases of unemancipated minors seeking an abortion were not deemed unconstitutional because the *dignity of the family* was invoked to permit some restrictions on the *dignity of the woman* and her unrestricted right to an abortion. This latter account of dignity, Scarffe argues, departs from the dominant Kantian account of dignity as individual autonomy and choice, replacing it with a different account of dignity more akin to that derived from its etymology, relating to rank and social status. Since dignity is a polyvalent rather than a static moral concept, its meaning can change as it is invoked in different ways. We see this again in *Planned Parenthood v. Casey*, 505 U.S. 833 (1992) when, despite Kennedy’s position on dignity in *Akron*, he joined other justices to once again reaffirm the meaning of dignity as an individual right to make a personal choice over whether to have a child.

In other legal cases concerning not only abortion but also LGBTQIA+ rights, Scarffe’s analysis highlights how dignity is invoked in ways that slide the scale of government interference. Dignity might be called upon to restrict personal autonomy in some instances, justifying legal interference on account of some other’s competing dignity claims, or cases may appeal to dignity as an egalitarian call, noting how “dignitary wounds” may be inflicted on those who are not granted equal dignity before the law. Appealing to dignity as a concept in these cases stresses the *contextual* importance of dignity as a legal and moral concept, one that serves a function in a society that is striving to find a fair and reasonable middle ground between competing demands to respect the rights — and dignity — of the individual, and to protect the rights — and dignity — of others in the wider community.

Yet the affirmation and denial of dignity to different legal subjects have not always been a “balancing act.” Decisions to respect dignity are historically and contextually located, grounded in wider sets of norms and assumptions about the kinds of people whose dignity the law should protect, and those who are not afforded dignity, or whose dignity the law should restrict. It would be amiss to simply describe dignity as an elusive or polyvalent concept when it is clear that it is a politically laden one; a concept that has been used as a tool for social exclusion, to deny persons sovereignty, and to strip them of their rights to legal personhood.

The myth of the egalitarianization of dignity, rooted in theological accounts of sovereignty’s extension to all persons, has been spread through Christian humanism. Dignity, as that bestowed upon sovereignty by grace, is also said to be bestowed upon the sovereignty of individual persons who comprise the body of the king and are thought, as *imagio dei*, to be made in the image of God.^2^ This conception of dignity has particularly informed some Christian attitudes toward the person, such as those found in the Personhood Movements that use strategies to analogize the historic depersonalization of slaves and the contemporary depersonalization of the fetus.

Human rights frameworks emerging post-World War II echoed Christian humanist appeals to respond to hierarchical conceptions of dignity inherent in fascism. As James Whitman (2003) notes,^3^ dignity in the era after the war had been pronounced as a universalizing strategy to protect persons from the kinds of hierarchized divisions of persons that emerged in Nazi Germany. Appealing to egalitarian conceptions of dignity for all, in contrast to particularistic claims to dignity as rank, however, ignores how the latter accounts have been bound to the former through the very articulation of personhood and sovereignty. Far from dignity emerging as a universalizing language post-WWII, the Nazi occupation itself drew on the same kinds of universalizing language of dignity to differentiate between persons. Nazism, as Whitman notes, did not consider itself to be hierarchically differentiating persons *on the basis of* dignity, but rather because those without dignity were also exempt from personhood on all counts. The question for Nazism was not whether a person had dignity because they had honour or worth, but whether one was even considered a person at all.

Universal appeals to dignity have thus been restricted in time and place on account of whether one belongs to the very category of human person. Dignity as a concept is used in different ways to determine *who counts* as a member of political society, and thus who is regarded as a legal person. I agree with Scarffe that it is evident in law that concepts are often invoked in different ways. However, I would question whether dignity in *Akron* really does depart from its conceptualisation in *Thornburgh* and *Casey.* Both accounts frame dignity as a concept that protects one group’s sovereignty over another. In each case, the law protects competing versions of sovereignty based on the sliding scale of personhood. The reality of the legal clashes between competing personhood in abortion cases, and in denial of personhood in cases of women’s rights, LGBTQIA+ rights, indigenous rights, disability rights and so on, suggest that dignity is bound to the broader question of who is said to have personhood and sovereignty, and thus protection and rights under the law, and who is excluded from our legal community.
